# Follow-Up Imaging Guidelines for Patients with Stage III Unresectable NSCLC: Recommendations Based on the PACIFIC Trial

**DOI:** 10.3390/curroncol30040289

**Published:** 2023-03-29

**Authors:** Jenny J. Ko, Shantanu Banerji, Normand Blais, Anthony Brade, Cathy Clelland, Devin Schellenberg, Stephanie Snow, Paul Wheatley-Price, Ren Yuan, Barbara Melosky

**Affiliations:** 1 Department of Medical Oncology, BC Cancer—Abbotsford, 32900 Marshall Road, Abbotsford, BC V2S 0C2, Canada; 2 CancerCare Manitoba Research Institute, CancerCare Manitoba, University of Manitoba, 675 McDermot Avenue, Winnipeg, MB R3E 0V9, Canada; 3 Centre Hospitalier de l’Université de Montréal, University of Montréal, 1051 Rue Sanguinet, Montréal, QC H2X 3E4, Canada; 4 Peel Regional Cancer Centre, Credit Valley Hospital, 2200 Eglinton Avenue W, Mississauga, ON L5M 2N1, Canada; 5 Primary Care, BC Cancer Primary Care Program, 600 W 10th Avenue, Vancouver, BC V5Z 4E6, Canada; 6 Department of Radiation Oncology, BC Cancer—Surrey Centre, 13750 96 Avenue, Surrey, BC V3V 1Z2, Canada; 7 QEII Health Sciences Centre, Dalhousie University, 5788 University Avenue, Halifax, NS B3H 1V8, Canada; 8 Department of Medicine, Ottawa Hospital Research Institute, The Ottawa Hospital, University of Ottawa, 501 Smyth Road, Ottawa, ON K1H 8L6, Canada; 9 Department of Diagnostic Imaging, BC Cancer–Vancouver Centre, 600 W 10th Avenue, Vancouver, BC V5Z 4E6, Canada; 10 Department of Medical Oncology, BC Cancer–Vancouver Centre, 600 W 10th Avenue, Vancouver, BC V5Z 4E6, Canada

**Keywords:** non-small cell lung cancer, stage III, follow-up imaging, guidelines, durvalumab

## Abstract

The PACIFIC trial showed a survival benefit with durvalumab through five years in stage III unresectable non-small cell lung cancer (NSCLC). However, optimal use of imaging to detect disease progression remains unclearly defined for this population. An expert working group convened to consider available evidence and clinical experience and develop recommendations for follow-up imaging after concurrent chemotherapy and radiation therapy (CRT). Voting on agreement was conducted anonymously via online survey. Follow-up imaging was recommended for all suitable patients after CRT completion regardless of whether durvalumab is received. Imaging should occur every 3 months in Year 1, at least every 6 months in Year 2, and at least every 12 months in Years 3–5. Contrast computed tomography was preferred; routine brain imaging was not recommended for asymptomatic patients. The medical oncologist should follow-up during Year 1 of durvalumab therapy, with radiation oncologist involvement if pneumonitis is suspected; medical and radiation oncologists can subsequently alternate follow-up. Some patients can transition to the family physician/community primary care team at the end of Year 2. In Years 1–5, patients should receive information regarding smoking cessation, comorbidity management, vaccinations, and general follow-up care. These recommendations provide guidance on follow-up imaging for patients with stage III unresectable NSCLC whether or not they receive durvalumab consolidation therapy.

## 1. Introduction

In 2020, lung cancer was the second most commonly diagnosed malignancy, with more than 2.2 million cases reported worldwide [1]. The disease was also the leading cause of cancer-related mortality, contributing to more than 1.7 million deaths globally [1]. Non-small cell lung cancer (NSCLC) is the most common disease subtype, representing approximately 88% of all lung cancer cases [2]. At diagnosis, 19% to 26% of patients present with stage III NSCLC [3,4], a heterogenous stage characterized by a range of pathophysiological presentations, which, alongside variations in patient characteristics (e.g., performance status (PS)), impact decisions related to treatment [5,6,7]. 

After diagnosis, patients with stage III NSCLC are evaluated for curative-intent therapy, which is typically multi-modal and highly case-specific [5,6,7]. Among the estimated 87% of patients who present with unresectable disease [3], those who are fit (Eastern Cooperative Oncology Group Performance Status (ECOG PS) 0–1), have adequate lung function, and have disease that can be radically treated with radiation are recommended to receive concurrent platinum-based chemotherapy and radiation therapy (CRT) [5,6,7,8,9]. For patients who do not progress after CRT, one year of consolidation therapy with the programmed death-ligand 1 (PD-L1) inhibitor durvalumab (IMFINIZI^®^) has become the standard of care worldwide based on the primary results of the Phase III PACIFIC trial [5,8,9,10,11,12]. Long-term outcomes from PACIFIC were recently reported, showing that the survival benefits of CRT + durvalumab were durable through five years of follow-up (median overall survival (mOS): 47.5 vs. 29.1 months with CRT alone; 5-year OS: 42.9% vs. 33.4%) [13].

The favorable long-term results of the PACIFIC trial have raised questions regarding optimal follow-up practices for patients with stage III unresectable NSCLC who receive treatment with CRT and durvalumab. Patient follow-up typically involves medical history, physical examination, radiographic imaging (usually computed tomography [CT]), and in some cases, serum biomarker evaluation [14,15]. These assessments are conducted with the goals of early detection of disease recurrence in asymptomatic patients and initiation of appropriate therapy [6,16,17,18,19]. Timely initiation of curative-intent treatment, radiotherapy (for isolated oligometastatic disease), or broader systemic therapy can potentially improve clinical outcomes [6,14,15,16,20]; however, the survival impact of varying imaging frequency remains unclear [17,21,22,23]. A prospective, randomized-controlled trial is needed to fully understand optimal imaging procedures, particularly among patients receiving CRT + durvalumab [24].

As a result of the limited evidence informing optimal follow-up practices, recommendations for patients with stage III unresectable NSCLC who have undergone curative-intent therapy vary across published guidelines, including those available in Canada [6,8,9,14,15,25]. One of the most recent and referenced recommendations, the 2020 American Society of Clinical Oncology (ASCO) guidelines, largely focuses on patients with resected stage I–III NSCLC, a population with a very different relapse risk and salvage therapy options than patients with stage III unresectable disease who have received CRT [14,26,27]. Furthermore, these guidelines do not yet consider the long-term survival findings for durvalumab in the PACIFIC trial [13]. Not surprisingly, differences across guidelines have led to variations in clinical practice. In 2021, the BC Cancer Lung Group conducted a regional survey to assess current follow-up practices for patients with stage III unresectable NSCLC, the results of which showed highly variable imaging practices across the province [28].

Given the recent results of the PACIFIC trial, variations in follow-up guidelines and practices worldwide, and patterns of relapse in the stage III unresectable NSCLC population, updated guidance is needed to inform best practices that will maximize patient outcomes while additional clinical data are generated. Recognizing this need, preliminary guidelines were proposed by the BC Cancer Lung Group for British Columbia in 2021, which described imaging, clinician follow-up, and transition of care recommendations for patients with stage III unresectable NSCLC receiving CRT ± durvalumab [28]. A pan-Canadian multidisciplinary expert working group was subsequently convened with the aim of developing recommendations that could be implemented across Canada and globally. This publication summarizes the clinical questions that were discussed by the expert working group, the recommendations that were developed, and the key issues that should be considered when implementing these recommendations.

## 2. Materials and Methods

In January 2022, a pan-Canadian multidisciplinary expert working group was formed that included nine individuals: six medical oncologists (S.B., N.B., J.J.K., B.M., S.S., P.W.P.), two radiation oncologists (A.B., D.S.), and a family physician (C.C.). The experts were chosen to be geographically representative (i.e., from western Canada, Ontario, and eastern Canada), experts in medical or radiation oncology treatment in NSCLC, and/or experts in the provision of follow-up care in the community care setting. The number of experts was considered appropriate, as the literature indicates that inclusion of a minimum of 5 and a maximum of 12 experts falls within a reasonable range to assess consensus [29]. The designees of the consultation were J.J.K. and B.M. After development of the key recommendations, a radiologist (R.Y.) was consulted to provide specific guidance regarding imaging modalities, body regions for scanning, and available supporting evidence. 

The expert working group initially convened virtually via videoconference in January 2022 to discuss five preliminary clinical questions related to the imaging of patients with stage III unresectable NSCLC after curative-intent treatment with CRT and considering use of durvalumab as consolidation therapy. The clinical questions were derived from the findings of the 2021 BC Cancer Lung Group regional survey. The group discussed each question while considering the wording of the proposed BC Lung Cancer Management Manual [30], published evidence, and their own clinical practice experience. Comments on the wording of the questions, initial recommendations and their underlying rationale, and additional feedback were documented in a summary report. Using the discussion from the meeting, a refined set of clinical questions and formal recommendations were prepared for review and voting. The updated questions included the following:

(1) What timepoint should be used as a reference for initiation of imaging?
(2) What is the recommended frequency of imaging?
(3) What type of imaging should be used and which body regions should be assessed?
(4) Who should follow the patient?
(5) What other assessments or activities should be conducted?

In February 2022, the updated clinical questions and formal recommendations were provided to the expert working group via an online survey (Microsoft Forms, Microsoft Corporation, Washington, DC, USA) for review, voting, and provision of additional comments and considerations. Each expert provided their feedback individually without seeing the responses of others; voting on the recommendations occurred by indicating agreement or disagreement. Using an iterative process, the key recommendations and their underlying rationale were revised and recirculated among the experts until at least seven of nine advisors agreed with each recommendation. 

## 3. Results and Discussion 

### 3.1. Summary of Recommendations 

A summary of the recommendations and key considerations developed by the expert working group is presented in Table 1; additional details are provided below.

### 3.2. Detailed Recommendations

**CLINICAL QUESTION 1:** What timepoint should be used as a reference for initiation of imaging?

**Recommendation 1.1:** For all patients with stage III unresectable NSCLC, imaging should be performed after completion of CRT, regardless of whether durvalumab is received.

**Figure 1:**
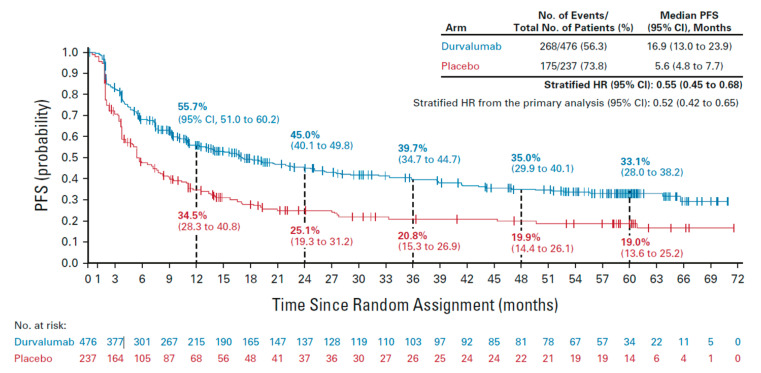
Progression-free survival in the ITT population of the PACIFIC trial. Vertical dashed lines indicate annual landmarks; the associated values represent the PFS rate at each timepoint. PFS was defined as the time from random assignment to the date of the first documented event of tumor progression or death in the absence of disease progression. Patients who had not progressed or died at the time of data cut-off were censored at the time of their last evaluable RECIST assessment; however, if a patient progressed or died after ≥2 missed visits, they were censored at the time of the latest evaluable RECIST assessment before the two missed visits. CI, confidence interval; HR, hazard ratio; RECIST, Response Evaluation Criteria in Solid Tumors; PFS, progression-free survival [13]. Figure used with permission from Wolters Kluwer Health, Inc. The Creative Commons license does not apply to this content. Use of the material in any format is prohibited without written permission from the publisher, Wolters Kluwer Health, Inc.

**Clinical Rationale:** Patients with stage III, unresectable NSCLC have the highest risk of disease relapse in the first year after completion of CRT independent of whether durvalumab consolidation therapy is received [13]. In the PACIFIC trial, rates of progression-free survival (PFS) were 55.7% with CRT + durvalumab and 34.5% with CRT alone at one year of follow-up, 45.0% and 25.1% at two years, 39.7% and 20.8% at three years, 35.0% and 19.9% at four years, and 33.1% and 19.0% at five years (Figure 1) [13]. Accordingly, for most patients, initiation of routine imaging and other assessments is imperative to monitor for signs and symptoms indicative of disease recurrence or progression such that timely and appropriate treatment can be provided [6,16,17,18]. Early detection of recurrent or metastatic disease may allow for early intervention and consideration of a broad range of management options [31,32].

**Figure 2:**
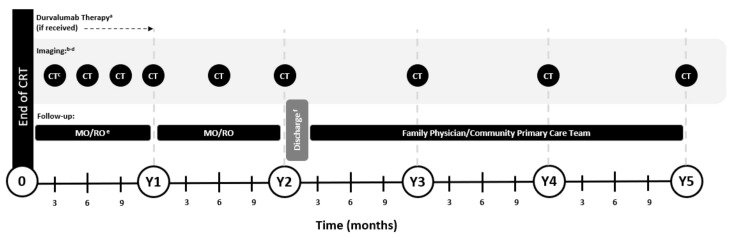
Recommendations for imaging and follow-up of patients with stage III, unresectable NSCLC after treatment with CRT ± durvalumab. Please refer to the text and Table 1 for additional considerations and caveats. ^a^ Durvalumab is approved for 1 year of consolidation therapy for patients who have not progressed after CRT; duration of therapy may vary depending on patient response and toxicity. ^b^ Baseline imaging is required after CRT and prior to durvalumab initiation to verify that NSCLC has not progressed. Imaging should occur Q3M until durvalumab therapy is stopped. ^c^ All CTs are chest/abdomen with contrast (if tolerated) through Year 5. ^d^ Baseline brain MRI should be completed in staging work-up before CRT. Subsequent brain imaging is only recommended in the event of symptom presentation; a low threshold of suspicion should prompt assessment. MRI is preferred over CT, if an option. ^e^ In Year 1 of durvalumab therapy, the RO (and possibly respirology) should be involved if associated pneumonitis is suspected; the MO and RO should alternate follow-up in Years 2 and 3 and throughout Years 1 to 3 for non-durvalumab patients. ^f^ Suitable patients only, see full text. CRT, chemotherapy + radiation therapy; CT, computed tomography; MO, medical oncologist; MRI, magnetic resonance imaging; NSCLC, non-small cell lung cancer; Q3M, every three months; RO, radiation oncologist; Y, year.

The expert working group recommended that regular imaging be performed after completion of CRT irrespective of reception of durvalumab therapy so as to facilitate consistent clinical practices across patients (Figure 2); it should be noted that patients who receive durvalumab remain on active treatment in Year 1. As stated in the ASCO guidelines, not all patients are amenable to imaging: some may be unwilling to proceed with subsequent therapy in the event that disease progression is identified, while others may not be clinical candidates for such treatment [14]. Regular imaging of these patients is unlikely to inform subsequent care and therefore may not be required. As stated by ASCO, age and PS should not preclude follow-up imaging, although discussion of the potential benefits and risks with patients is recommended [14].

**CLINICAL QUESTION 2:** What is the recommended frequency of imaging?

**Recommendation 2.1:** In Year 1 after CRT, all patients should undergo imaging every 3 months.

**Recommendation 2.2:** In Year 2, all patients should undergo imaging at least every 6 months.

**Recommendation 2.3:** In Years 3, 4, and 5, all patients should undergo imaging at least every 12 months.

**Clinical Rationale:** In the PACIFIC trial, tumor assessment scans (CT or MRI; RECIST 1.1) occurred at screening and then every two months in Year 1 or until disease progression [33]. Among patients with disease control after completion of durvalumab, imaging assessments were performed every three months until progressive disease was confirmed. As highlighted previously, the trial showed that regardless of whether durvalumab was received, patients with stage III, unresectable NSCLC who received curative-intent therapy had the highest risk of relapse in the first year after CRT (Figure 1). Other evidence similarly indicates that recurrence of NSCLC is generally highest during the first one-to-two years after treatment with curative intent [13,17,34,35]. 

Given these findings, the expert working group recommended that imaging frequency should vary over time in accordance with the year-over-year risk of progression after CRT and the need for close monitoring while patients are actively receiving durvalumab. As such, for patients proceeding with durvalumab consolidation therapy, imaging should first be conducted two-to-four weeks after completion of CRT and before initiation of durvalumab; subsequent imaging should occur every three months for the full duration of durvalumab therapy, which is typically one year (Figure 2) [11]. For patients not initiating durvalumab, imaging should be initiated three months after completion of CRT and continued every three months in Year 1. In Year 2, all patients should undergo imaging at least every six months; after Year 2, routine imaging was recommended at least every 12 months through the end of Year 5, after which time patients should continue to undergo regular clinical assessment. Routine imaging was not recommended after Year 5, as no data were available to support an additional benefit; furthermore, lung cancer screening trials have not typically included patients with a prior history of lung cancer. Still, ongoing imaging may be prudent depending on patient history and risk factors [36,37,38]. 

The expert working group underscored that all recommended timepoints are minimum frequencies that should be employed among patients without suspicion of recurrent disease; more frequent surveillance is warranted for patients with an elevated risk of or suspected progression. The experts recognized that the survival impact of more versus less frequent follow-up imaging remains equivocal: there is a paucity of high-quality evaluations of optimal imaging modalities, intervals, and durations of follow-up after definitive treatment [6,14,15]. Several studies have reported that although more frequent imaging detects asymptomatic recurrence of NSCLC at high rates, no survival benefit is observed [21,22,23]. Conversely, one retrospective study found that patients with symptomatic relapse had poorer mOS than those identified using surveillance imaging (23 vs. 36 months, respectively; *p* = 0.013) [17]. Given that newer treatment options are available for both oligometastatic and widespread disease, additional studies are needed—particularly in the stage III unresectable population—to assess the appropriate frequency of follow-up and the potential impact on survival outcomes.

**CLINICAL QUESTION 3:** What type of imaging should be used and which body regions should be assessed?

**Recommendation 3.1:** Contrast CT of the chest and upper abdomen should be used through the end of Year 5 after CRT.

**Recommendation 3.2**: Routine brain imaging is not recommended.

**Clinical Rationale:** In the PACIFIC trial, tumor assessment scans of the chest and abdomen (including the liver and adrenals) were conducted using contrast CT or MRI [33]. In both treatment arms, the most frequent site of disease recurrence was the lung (13.4% with durvalumab; 18.1% with placebo); other sites of recurrence included the lymph nodes (most common), brain, liver, bones, and adrenal glands [13]. As such, comprehensive imaging is imperative to identify new lesions or local progression among patients with stage III unresectable NSCLC.

The ASCO guidelines recommend use of CT with contrast in the first two years of surveillance, followed by low-dose CT in Years 3 to 5; however, these recommendations focus on patients with resected, earlier-stage (I–III) NSCLC who have a lower risk of disease recurrence than the unresectable stage III population [14]. In the unresectable stage III setting, the expert working group recommended standard-dose contrast CT of the chest and upper abdomen through the end of Year 5 (Figure 2). Imaging of these regions permits adequate assessment of the most frequent sites of recurrent disease, including the lung, thoracic lymph nodes, liver, adrenals, and thoracic spine [39]. Use of contrast-enhanced CT was recommended over unenhanced CT, as the former offers greater accuracy and reduced inter-reader variability in the identification of hilar lymph nodes, as well as reliable detection of mediastinal lymph nodes and abdominal progression [40]. In general, imaging of the chest including the upper abdomen (adrenal glands and liver) with intravenous contrast is a common and well-accepted CT protocol for lung cancer follow-up [41,42]. As patients with stage III disease have already received definitive doses of radiation, the risk of additional exposure to contrast CT was considered minimal. Moreover, there remains a paucity of evidence for use of low-dose CT during active surveillance among patients with lung cancer—available studies have primarily focused on lung cancer screening in high-risk populations. Prospective studies are needed to assess the sensitivity of this modality in the curative-intent setting of unresectable stage III NSCLC. Still, the expert working group recognized that intravenous contrast CT is contraindicated for some patients, such as those with a history of anaphylactic reaction to iodinated CT contrast media or who have severe kidney impairment [43,44,45].

The expert working group additionally recommended that at baseline, all suitable patients should have a brain MRI with contrast completed as part of the staging work-up for CRT [8,9]; however, after completion of CRT, routine brain imaging was not recommended. As noted in the ASCO guidelines, to date, no randomized clinical trials have evaluated use of brain MRI for surveillance in NSCLC [14]. Therefore, the guidelines do not recommend routine brain MRI in this setting. Still, at a median follow-up of 34.2 months in the PACIFIC trial, the brain was a common site of extra-thoracic recurrence: new brain lesions occurred in 6.5% of CRT + durvalumab-treated patients and 11.8% of those treated with CRT alone [13]. As such, although routine imaging was not recommended by the expert working group, a low threshold of suspicion of central nervous system symptoms should prompt assessment. When imaging is conducted, MRI was preferred over CT, where an option, given its higher sensitivity [46,47,48]. In the event that central nervous system metastases are identified, the patient should be referred to a radiation oncologist and/or neurosurgeon for consideration of an optimal management plan.

**CLINICAL QUESTION 4:** Who should follow the patient?

**Recommendation 4.1:** For patients who receive durvalumab, the medical oncologist should follow during Year 1 after CRT; the radiation oncologist should be involved if pneumonitis is suspected.

**Recommendation 4.2:** After Year 1, follow-up can alternate between the medical oncologist and radiation oncologist until transition to the family physician/community primary care team.

**Recommendation 4.3:** For patients who do not receive durvalumab, follow-up can alternate post-CRT between the medical oncologist and the radiation oncologist until transition to the family physician/community primary care team.

**Recommendation 4.4:** Some patients can be transitioned to the family physician/community primary care team at the end of Year 2 after completion of CRT.

**Clinical Rationale:** Throughout the course of follow-up, it is important to clearly define the responsible clinician(s) to ensure that monitoring for adverse events (AEs), imaging for disease recurrence and/or progression, and other assessments are completed on schedule and with accountability for subsequent patient needs. Responsible individual(s) may vary according to patient presentation, region, and/or healthcare center, as well as over time.

In Year 1 after CRT during active treatment with durvalumab, the expert working group recommended that the medical oncologist follow the patient; the radiation oncologist should be involved if pneumonitis is suspected, and in some centers, respirology would also be involved (Figure 2). This recommendation was based on the challenges encountered in distinguishing the etiology of pneumonitis as being related to radiation or immunotherapy [49]. At 14.5 months of follow-up in the PACIFIC trial, pneumonitis or radiation pneumonitis of any grade and cause was reported in 33.9% of durvalumab-treated patients; grade 3 events occurred in 3.4% [10]. The median time to onset of pneumonitis from treatment initiation was approximately 1.8 months; patients with this AE were more likely to be Asian or have mutations of the epidermal growth factor receptor gene [50].

After Year 1 of durvalumab therapy, the expert working group recommended that follow-up alternate between the medical and radiation oncologists until transition to the family physician/community primary care team; patients who do not receive durvalumab can receive alternating follow-up in Years 1 and 2 (Figure 2). Given the declining risk of disease recurrence over time and the fact that some patients reside far from their treating cancer center, the experts stated that suitable patients who have a reliable family physician/community primary care team can be transitioned at the end of Year 2 (i.e., one year after completion of durvalumab). It should be noted that this recommendation was made with caution: several experts raised concerns regarding the risk of delayed immunotherapy-related AEs that may present after completion of durvalumab. The experts indicated that transfer notes to the family physician/community primary care team should include clear instructions on follow-up imaging requirements. Furthermore, patients should be referred back to the medical and/or radiation oncologist immediately upon suspicion of disease recurrence or progression.

**CLINICAL QUESTION 5:** What other assessments or activities should be conducted?

**Recommendation 5.1:** Throughout Years 1 to 5 after CRT, patients should receive information on smoking cessation, comorbidity management, and relevant vaccinations, as applicable, and be encouraged to receive regular follow-up care for non-cancer conditions and general health concerns.

**Clinical Rationale:** In alignment with the ASCO guidelines [14], the expert working group underscored that clear communication and healthcare coordination play critical roles in the care of cancer survivors. Furthermore, the comprehensive care of these individuals should address both cancer-related and general care needs. These needs include not only ongoing surveillance for disease recurrence or progression, but also monitoring and management of psychosocial issues and chronic medical conditions, general health promotion, and disease prevention [14]. Community primary care teams should provide counselling on the importance of smoking cessation and vaccinations for influenza, Streptococcus pneumoniae, and COVID-19, among others. The implications of common comorbidities, such as chronic obstructive pulmonary disease, should also be discussed.

Throughout the course of treatment and follow-up of patients with stage III unresectable NSCLC, the family physician/community primary care team should be kept informed of the results of all clinical assessments and continue to provide care for non-cancer health, family, and psychosocial concerns. All patients should receive a summary of key health-related items for consideration and follow-up over the course of their disease. This summary should include information regarding the potential impact of cancer therapy on their health, including current comorbidities and the risk of new long-term complications.

## 4. Conclusions

The favorable five-year survival results of the PACIFIC trial of durvalumab have prompted reconsideration of best follow-up imaging practices for patients with stage III unresectable NSCLC. Until new data are available showing optimal imaging frequency and modality and a need for other assessments, these recommendations provide globally relevant direction for clinicians treating this patient population.

## Figures and Tables

**Table 1:** Summary of clinical questions, recommendations, and key considerations.

**Target Population**	Patients with curatively treated, stage III, unresectable NSCLC who complete CRT ± consolidation therapy with durvalumab as per the PACIFIC trial and are without clinical suspicion of recurrent disease.	
**Target Audience**	Medical, surgical, and radiation oncologists; oncology nurses and physician assistants; pulmonologists, radiologists; family physician/community primary care team; and patients.	
**Note**	Patient history and physical examination should occur at regular intervals in all years.	
**Clinical Question**	**Recommendation**	**Key Considerations**
1. What timepoint should be used as a reference for initiation of imaging?	**Recommendation 1.1**: For all patients with stage III unresectable NSCLC, imaging should be performed after completion of CRT, regardless of whether durvalumab is received.	Regular imaging was recommended whether or not durvalumab consolidation therapy is received to facilitate consistent integration into clinical practice. Imaging should occur among patients for whom intervention would be appropriate in the event of relapse.
2. What is the recommended frequency of imaging?	**Recommendation 2.1**: In Year 1 after CRT, all patients should undergo imaging every 3 months. **Recommendation 2.2**: In Year 2, all patients should undergo imaging at least every 6 months. **Recommendation 2.3**: In Years 3, 4, and 5, all patients should undergo imaging at least every 12 months.	The highest risk of disease relapse occurs in the first year after CRT, regardless of whether durvalumab is received. For patients receiving durvalumab, imaging should begin 2–4 weeks after completion of CRT and before durvalumab initiation; imaging should occur Q3M during durvalumab therapy. For patients not initiating durvalumab, imaging should begin 3 months after completion of CRT.
3. What type of imaging should be used and which body regions should be assessed?	**Recommendation 3.1**: Contrast CT of the chest and upper abdomen should be used through the end of Year 5 after CRT. **Recommendation 3.2**: Routine brain imaging is not recommended.	Non-contrast CT is inadequate to identify mediastinal or abdominal disease progression. Studies of low-dose CT have not included patients with prior lung cancer; prospective studies are needed to properly inform use. Baseline brain MRI should have been completed as part of staging work-up before CRT. A low threshold of suspicion of CNS symptoms should prompt assessment; brain MRI is preferred over CT, when an option, given higher sensitivity.
4. Who should follow the patient?	**Recommendation 4.1**: For patients who receive durvalumab, the medical oncologist should follow during Year 1 after CRT; the radiation oncologist should be involved if pneumonitis is suspected. **Recommendation 4.2**: After Year 1, follow-up can alternate between the medical oncologist and radiation oncologist until transition to the family physician/community primary care team. **Recommendation 4.3**: For patients who do not receive durvalumab, follow-up can alternate between the medical oncologist and the radiation oncologist post-CRT until transition to the family physician/community primary care team. **Recommendation 4.4**: Some patients can be transitioned to the family physician/community primary care team after Year 2 following completion of CRT.	The specific cause of pneumonitis can be difficult to identify in Year 1 after CRT during durvalumab therapy; the radiation oncologist and/or respirologist should be involved in identification. Patients should only be transitioned if they have reliable family physician/community primary care team contact and follow-up. Transfer notes to the family physician/community primary care team should include clear directions on imaging requirements. Patients should be referred back to the medical oncologist and/or radiation oncologist immediately upon suspicion of disease recurrence or progression.
5. What other assessments or activities should be conducted?	**Recommendation 5.1**: Throughout Years 1 to 5 after CRT, patients should receive information on smoking cessation, comorbidity management, and relevant vaccinations, as applicable, and be encouraged to receive regular follow-up care for non-cancer conditions and general health concerns.	The family physician/community primary care team should be involved throughout follow-up to provide ongoing patient support in terms of regular health check-ups and screening activities (e.g., for non-cancer conditions). All patients should receive a summary of health-related items for consideration, including their care plan and post-active treatment information.

CNS, central nervous system; CRT, chemotherapy + radiation therapy; CT, computed tomography; MRI, magnetic resonance imaging; Q3M, every three months.

## Data Availability

Data sharing not applicable. No new data were created or analyzed in this study. Data sharing is not applicable to this article.

## References

[1] Sung H., Ferlay J., Siegel R.L., Laversanne M., Soerjomataram I., Jemal A., Bray F. (2021). Global Cancer Statistics 2020: GLOBOCAN estimates of incidence and mortality worldwide for 36 cancers in 185 countries. CA Cancer J. Clin..

[2] Canadian Cancer Statistics Advisory Committee Canadian Cancer Statistics: A 2020 Special Report on Lung Cancer. Cancer.ca/Canadian-Cancer-Statistics-2020-EN.

[3] Seung S.J., Hurry M., Walton R.N., Evans W.K. (2020). Retrospective cohort study of unresectable stage III non-small-cell lung cancer in Canada. Curr. Oncol..

[4] Ganti A.K., Klein A.B., Cotarla I., Seal B., Chou E. (2021). Update of incidence, prevalence, survival, and initial treatment in patients with non–small cell lung cancer in the US. JAMA Oncol..

[5] Robinson A., Vella E.T., Ellis P.M., Goffin R., Hanna W., Maziak D., Swaminath A., Ung Y.C., the Lung Cancer Disease Site Group Ontario Health. Cancer Care Ontario. Recommendations for the Treatment of Patients with Clinical Stage III Non-Small Cell Lung Cancer: Endorsement of the 2019 National Institute for Health and Care Excellence Guidance and the 2018 Society for Immunotherapy of Cancer Guidance. 27 April 2020. https://www.cancercareontario.ca/en/file/54406/download?token=oMLjCMXY.

[6] Provincial Health Services Authority BC Cancer. Treatment of Locally Advanced Non-Small Cell Lung Cancer. http://www.bccancer.bc.ca/books/lung/management/non-small-cell-lung-cancer-nsclc/combined-modality-therapy-for-unresectable-stage-iii.

[7] Brade A., Jao K., Yu S., Cheema P., Doucette S., Christofides A., Schellenberg D. (2021). A Canadian perspective on the challenges for delivery of curative-intent therapy in stage III unresectable non-small cell lung cancer. Curr. Oncol..

[8] National Comprehensive Cancer Network Clinical Practice Guidelines in Oncology: Non-Small Cell Lung Cancer. Version 1.2022. https://www.nccn.org.

[9] Postmus P.E., Kerr K.M., Oudkerk M., Senan S., Waller D.A., Vansteenkiste J., Escriu C., Peters S., on behalf of the ESMO Guidelines Committee ESMO Early and Locally Advanced Non-Small-Cell Lung Cancer (NSCLC) Guideline. http://interactiveguidelines.esmo.org/esmo-web-app/gl_toc/index.php?GL_id=46.

[10] Antonia S.J., Villegas A., Daniel D., Vicente D., Murakami S., Hui R., Yokoi T., Chiappori A., Lee K.H., de Wit M. (2017). Durvalumab after chemoradiotherapy in stage III non–small-cell lung cancer. N. Engl. J. Med..

[11] AstraZeneca Product Monograph: IMFINZI(R) (Durvalumab for Injection). Date of Revision: 21 September 2021. https://www.astrazeneca.ca/content/dam/az-ca/downloads/productinformation/imfinzi-product-monograph-en.pdf.

[12] Daly M.E., Singh N., Ismaila N., Antonoff M.B., Arenberg D.A., Bradley J., David E., Detterbeck F., Früh M., Gubens M.A. (2022). Management of stage III non-small-cell lung cancer: ASCO guideline. J. Clin. Oncol..

[13] Spigel D.R., Faivre-Finn C., Gray J.E., Vicente D., Planchard D., Paz-Ares L., Vansteenkiste J.F., Garassino M.C., Hui R., Quantin X. (2022). Five-year survival outcomes from the PACIFIC trial: Durvalumab after chemoradiotherapy in stage III non-small-cell lung cancer. J. Clin. Oncol..

[14] Schneider B.J., Ismaila N., Aerts J., Chiles C., Daly M.E., Detterbeck F.C., Hearn J.W.D., Katz S.I., Leighl N.B., Levy B. (2020). Lung cancer surveillance after definitive curative-intent therapy: ASCO guideline. J. Clin. Oncol..

[15] Ung Y.C., Souter L.H., Darling G., Dobranowski J., Donohue L., Leighl N., Ellis P.M., the Lung Cancer Follow-up Expert Panel Cancer Care Ontario. Follow-Up and Surveillance of Curatively Treated Lung Cancer Patients. 29 August 2014. https://www.cancercareontario.ca/sites/ccocancercare/files/guidelines/summary/pebc26-3s.pdf.

[16] Ho Q.-A., Harandi N.K., Daly M.E. (2017). Clinical impact of frequent surveillance imaging in the first year following chemoradiation for locally advanced non-small-cell lung cancer. Clin. Lung Cancer.

[17] Grass G.D., Naghavi A.O., Abuodeh Y.A., Perez B.A., Dilling T.J. (2019). Analysis of relapse events after definitive chemoradiotherapy in locally advanced non-small-cell lung cancer patients. Clin. Lung Cancer.

[18] Hall H., Tocock A., Burdett S., Fisher D., Ricketts W.M., Robson J., Round T., Gorolay S., MacArthur E., Chung D. (2021). Association between time-to-treatment and outcomes in non-small cell lung cancer: A systematic review. Thorax.

[19] Palma D.A., Olson R., Harrow S., Gaede S., Louie A.V., Haasbeek C., Mulroy L., Lock M., Rodrigues G.B., Yaremko B.P. (2019). Stereotactic ablative radiotherapy versus standard of care palliative treatment in patients with oligometastatic cancers (SABR-COMET): A randomised, phase 2, open-label trial. Lancet.

[20] Hearn J.W., Videtic G.M., Djemil T., Stephans K.L. (2014). Salvage stereotactic body radiation therapy (SBRT) for local failure after primary lung SBRT. Int. J. Radiat. Oncol. Biol. Phys..

[21] Benamore R., Shepherd F.A., Leighl N., Pintilie M., Patel M., Feld R., Herman S. (2007). Does intensive follow-up alter outcome in patients with advanced lung cancer?. J. Thor. Oncol..

[22] McMurry T.L., Stukenborg G.J., Kessler L.G., Colditz G.A., Wong M.L., Francescatti A.B., Jones D.R., Schumacher J.R., Greenberg C.C., Chang G.J. (2018). More frequent surveillance following lung cancer resection is not associated with improved survival: A nationally representative cohort study. Ann. Surg..

[23] Calman L., Beaver K., Hind D., Lorigan P., Roberts C., Lloyd-Jones M. (2011). Survival benefits from follow-up of patients with lung cancer: A systematic review and meta-analysis. J. Thorac. Oncol..

[24] Dyer B.A., Daly M.E. (2017). Surveillance imaging following definitive radiotherapy for non-small cell lung cancer: What is the clinical impact?. Semin. Oncol..

[25] Canadian Association of Thoracic Surgeons Follow-Up and Surveillance—Curatively-Treated Lung Cancer. https://www.canadianthoracicsurgeons.ca/2018/08/28/surveillance/.

[26] Jazieh A.R., Onal H.C., Tan D.S.W., Soo R.A., Prabhash K., Kumar A., Huggenberger R., Robb S., Cho B.-C. (2021). Real-world treatment patterns and clinical outcomes in patients with stage III NSCLC: Results of KINDLE, a multicountry observational study. J. Thorac. Oncol..

[27] Pignon J.P., Tribodet H., Scagliotti G.V., Douillard J.Y., Shepherd F.A., Stephens R.J., Dunant A., Torri V., Rosell R., Seymour L. (2008). Lung adjuvant cisplatin evaluation: A pooled analysis by the LACE Collaborative Group. J. Clin. Oncol..

[28] Ko J.J., Melosky B. (2022). Personal communication.

[29] Kea B., Sun B.C.-A. (2015). Consensus development for healthcare professionals. Intern. Emerg. Med..

[30] Provincial Health Services Authority BC Cancer. Lung-Management. http://www.bccancer.bc.ca/health-professionals/clinical-resources/cancer-management-manual/lung/lung#Management.

[31] Soria J.-C., Ohe Y., Vansteenkiste J., Reungwetwattana T., Chewaskulyong B., Lee K.H., Dechaphunkul A., Imamura F., Nogami N., Kurata T. (2017). Osimertinib in untreated EGFR-mutated advanced non–small-cell lung cancer. N. Engl. J. Med..

[32] Shields M.D., Marin-Acevedo J.A., Pellini B. (2021). Immunotherapy for advanced non–small cell lung cancer: A decade of progress. Am. Soc. Clin. Oncol. Educ. Book.

[33] Antonia S.J., Villegas A., Daniel D., Vicente D., Murakami S., Hui R., Kurata T., Chiappori A., Lee K.H., de Wit M. (2018). Trial protocol for: Overall survival with durvalumab after chemoradiotherapy in stage III NSCLC. N. Engl. J. Med..

[34] Lou F., Sima C.S., Rusch V.W., Jones D.R., Huang J. (2014). Differences in patterns of recurrence in early-stage versus locally advanced non-small cell lung cancer. Ann. Thorac. Surg..

[35] Sugimura H., Yang P. (2006). Long-term survivorship in lung cancer: A review. Chest.

[36] de Koning H.J., van der Aalst C.M., de Jong P.A., Scholten E.T., Nackaerts K., Heuvelmans M.A., Lammers J.-W.J., Weenink C., Yousaf-Khan U., Horeweg N. (2020). Reduced lung-cancer mortality with volume CT screening in a randomized trial. N. Engl. J. Med..

[37] Tammemägi M.C., Ruparel M., Tremblay A., Myers R., Mayo J., Yee J., Atkar-Khattra S., Yuan R., Cressman S., English J. (2022). USPSTF2013 versus PLCOm2012 lung cancer screening eligibility criteria (International Lung Screening Trial): Interim analysis of a prospective cohort study. Lancet Oncol..

[38] National Lung Screening Trial Research Team (2011). Reduced lung-cancer mortality with low-dose computed tomographic screening. N. Engl. J. Med..

[39] Johnson B.E. (1998). Second lung cancers in patients after treatment for an initial lung cancer. J. Natl. Cancer Inst..

[40] Takahashi M., Nitta N., Takazakura R., Nagatani Y., Ushio N., Murata K. (2008). Detection of mediastinal and hilar lymph nodes by 16-row MDCT: Is contrast material needed?. Eur. J. Radiol..

[41] García-Garrigós E., Arenas-Jiménez J.J., Sánchez-Payá J. (2018). Best protocol for combined contrast-enhanced thoracic and abdominal CT for lung cancer: A single-institution randomized crossover clinical trial. AJR Am. J. Roentgenol..

[42] Bhalla A.S., Das A., Naranje P., Irodi A., Raj V., Goyal A. (2019). Imaging protocols for CT chest: A recommendation. Indian J. Radiol. Imaging.

[43] Huynh K., Baghdanian A.H., Baghdanian A.A., Sun D.S., Kolli K.P., Zagoria R.J. (2020). Updated guidelines for intravenous contrast use for CT and MRI. Emerg. Radiol..

[44] Caraiani C., Petresc B., Dong Y., Dietrich C.F. (2019). Contraindications and adverse effects in abdominal imaging. Med. Ultrason..

[45] American College of Radiology (2021). ACR Manual on Contrast Media.

[46] Yokoi K., Kamiya N., Matsuguma H., Machida S., Hirose T., Mori K., Tominaga K. (1999). Detection of brain metastasis in potentially operable non-small cell lung cancer: A comparison of CT and MRI. Chest.

[47] Ellingson B.M., Bendszus M., Boxerman J., Barboriak D., Erickson B.J., Smits M., Nelson S.J., Gerstner E., Alexander B., Goldmacher G. (2015). Consensus recommendations for a standardized brain tumor imaging protocol in clinical trials. Neuro-Oncology.

[48] Kuhn M.J., Hammer G.M., Swenson L.C., Youssef H.T., Gleason T.J. (1994). MRI evaluation of “solitary” brain metastases with triple-dose gadoteridol: Comparison with contrast-enhanced CT and conventional-dose gadopentetate dimeglumine MRI studies in the same patients. Comput. Med. Imaging Graph..

[49] Naidoo J., Nishino M., Patel S.P., Shankar B., Rekhtman N., Illei P., Camus P. (2020). Immune-related pneumonitis after chemoradiotherapy and subsequent immune checkpoint blockade in unresectable stage III non–small-cell lung cancer. Clin. Lung Cancer.

[50] Vansteenkiste J., Naidoo J., Faivre-Finn C., Özgüroğlu M., Villegas A., Daniel D., Murakami S., Hui R., Lee K., Cho B.C. (2018). MA05. 02 PACIFIC subgroup analysis: Pneumonitis in stage III, unresectable NSCLC patients treated with durvalumab vs. placebo after CRT. J. Thorac. Oncol..

